# Supplementary material for: Associations of Biopterins and ADMA with Vascular Function in Peripheral Microcirculation from Patients with Chronic Kidney Disease
Source: Int J Mol Sci. 2023 Mar 15;24(6):5582. doi: 10.3390/ijms24065582 (PMC10056709; doi:10.3390/ijms24065582)
Supplement: Supplementary file 1 [file ijms-24-05582-s001.zip › ijms-2273569-supplementary.pdf]

Associations of Biopterins and ADMA with Vascular Function in Peripheral Microcirculation from Patients with Chronic Kidney Disease

Authors: Samsul Arefin[1], Lars Löfgren[2], Peter Stenvinkel[1], Anna B. Granqvist[2] and Karolina Kublickiene[1,*]

[1] Department of Clinical Science, Intervention & Technology, Division of Renal Medicine, Karolinska Institutet, 141 86 Stockholm, Sweden

[2] Early CVRM, BioPharmaceuticals R&D, AstraZeneca, 431 50 Mölndal, Sweden

* Correspondence: karolina.kublickiene@ki.se

Figure S1: Endothelium-dependent vasodilation reflected by reactive hyperemia index (RHI) (A) and stiffness reflected by augmentation index (AI@75) (B) between control, CKD-3 and CKD-5 groups. Data are expressed as median (interquartile range); significances were assessed by Mann-Whitney U test. P<0.05. CKD-3= chronic kidney disease stage 3; CKD-5= chronic kidney disease stage 5.

Figure S2: Contribution of NO and EDHF in controls and CKD-5. Data are expressed as median (interquartile range); significances were assessed by Mann-Whitney U test; NO =Nitric oxide; EDHF = endothelium-derived hyperpolarizing factor; CKD-5= chronic kidney disease stage 5

Figure S3: Concentration response curve to bradykinin (BK) induced relaxation in arteries before and after indomethacin inhibition in controls (A) and ESKD (B) participants. Data are expressed as mean ± SEM; significances were assessed by two-way ANOVA; PSS= physiological salt solution; ESKD= end stage kidney disease.

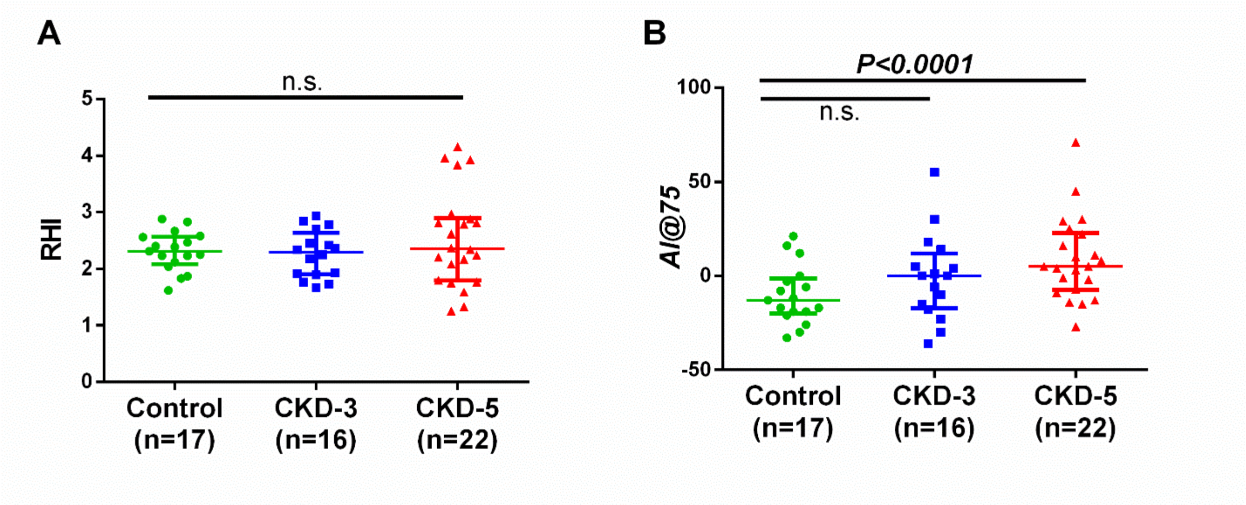

Figure S1: Endothelium-dependent vasodilation reflected by reactive hyperemia index (RHI) (A) and stiffness reflected by augmentation index (AI@75) (B) between control, CKD-3 and CKD-5 groups. Data are expressed as median (interquartile range); significances were assessed by Mann-Whitney U test. $P<0.05$. CKD-3= chronic kidney disease stage 3; CKD-5= chronic kidney disease stage 5.

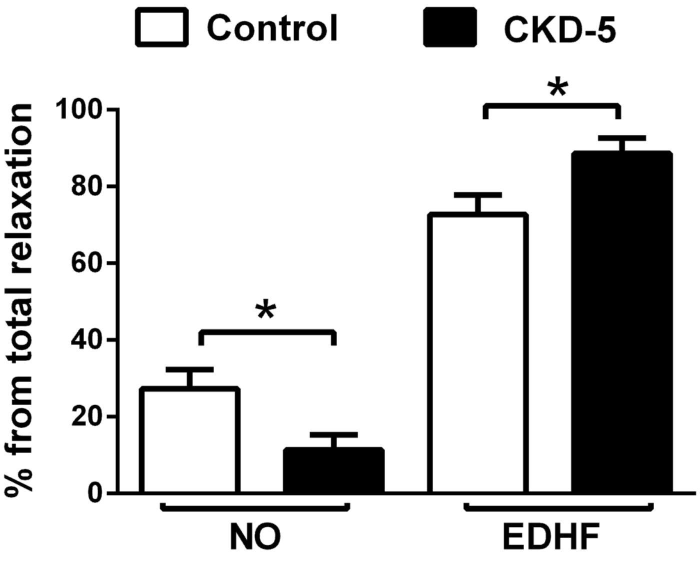

Figure S2: Contribution of NO and EDHF in controls and CKD-5participants. Data are expressed as median (interquartile range); significances were assessed by Mann-Whitney U test; NO =Nitric oxide; EDHF = endothelium-derived hyperpolarizing factor; CKD-5= chronic kidney disease stage 5

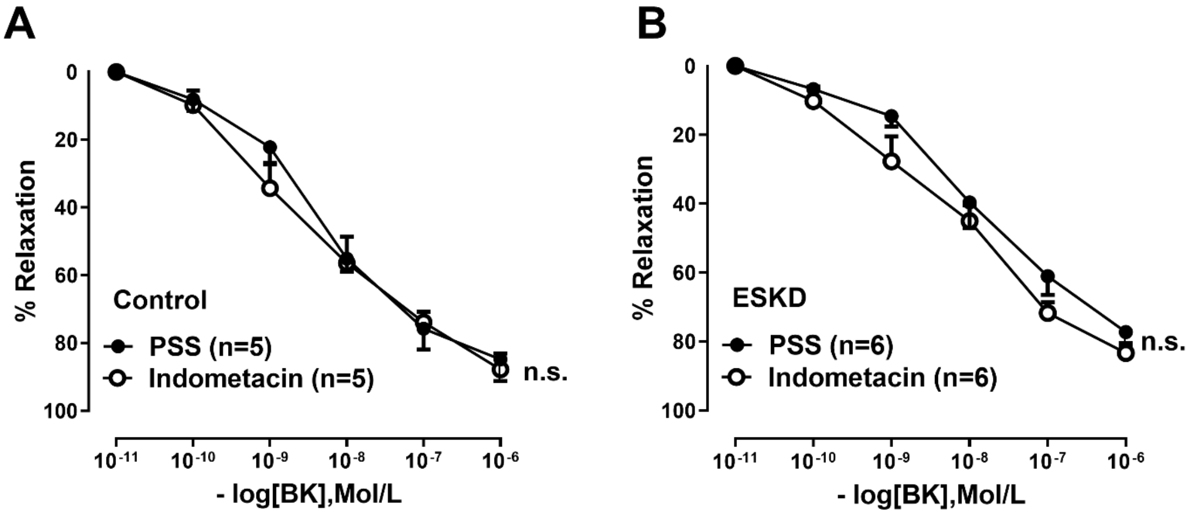

Figure S3: Concentration response curve to bradykinin (BK) induced relaxation in arteries before and after indomethacin inhibition in controls (A) and ESKD (B) participants. Data are expressed as mean ± SEM; significances were assessed by two-way ANOVA; PSS= physiological salt solution; ESKD= end stage kidney disease.
